# Gastric Foveolar‐Type Hyperplastic Polyp of the Duodenum With *GNAS* and *KRAS* Mutations: A Potential Precursor to Neoplasia

**DOI:** 10.1002/deo2.70207

**Published:** 2025-09-15

**Authors:** Kenji Yamazaki, Ryoji Kushima, Noritaka Ozawa, Haruka Koizumi, Saeka Hayashi, Atsushi Soga, Koji Yamashita, Shogo Shimizu, Masahito Shimizu

**Affiliations:** ^1^ Department of Gastroenterology Gifu Prefectural General Medical Center Gifu Japan; ^2^ Department of Gastroenterology Gifu University School of Medicine Gifu Japan; ^3^ Department of Pathology Shiga University of Medical Science Otsu Shiga Japan

**Keywords:** duodenum, gastric foveolar‐type hyperplastic polyp, *GNAS*, *KRAS*, non‐ampullary duodenal epithelial tumors

## Abstract

We report a gastric foveolar‐type hyperplastic polyp of the duodenum harboring mutations in the *GNAS* and *KRAS* genes. The lesion was incidentally detected during a routine upper gastrointestinal endoscopy in a man in his 50s. It appeared as a 10‐mm elevated lesion located at the superior duodenal angle. The surrounding mucosa showed no endoscopic evidence of gastric foveolar metaplasia (GFM) or heterotopic gastric mucosa (HGM). Endoscopic mucosal resection was performed as diagnostic treatment. Histopathology showed a diffuse, monotonous proliferation of gastric foveolar‐type epithelial cells without cytological dysplasia, and immunohistochemical analysis showed diffuse positivity for MUC5AC. Genetic analysis revealed activating mutations in both the *GNAS* and *KRAS* genes. Based on these findings, the final diagnosis was a gastric foveolar‐type hyperplastic polyp harboring *GNAS* and *KRAS* mutations. Such mutations have also been reported in pyloric gland adenomas and gastric‐type duodenal adenocarcinomas. Proximal non‐ampullary duodenal epithelial tumors are often linked to a gastric‐type mucin phenotype with higher malignant potential. Potential precursor lesions for carcinomas proximal to the ampulla include GFM and HGM. This case supports the hypothesis that some duodenal lesions with the gastric‐mucin phenotype may harbor molecular alterations typically associated with neoplastic processes despite their non‐neoplastic appearance, suggesting a potential role as precursors to neoplasia.

## 1 Introduction

Gastric foveolar metaplasia (GFM) and heterotopic gastric mucosa (HGM) are generally considered to represent reactive or congenital duodenal changes. However, recent studies have revealed that some of these lesions, even without cytological dysplasia, may harbor somatic mutations associated with neoplasia, particularly in the *GNAS* and *KRAS* genes [1]. These findings suggest that such lesions may represent precursors to non‐ampullary duodenal epithelial tumors (NADETs) with a gastric‐type mucin phenotype (G‐type) [1, 2], hereafter referred to as G‐type NADETs. A recent series of NADETs showed that G‐type lesions are uncommon but frequently harbor *GNAS* mutations, with *KRAS* mutations detected in a subset; in contrast, intestinal‐type tumors more often exhibit *APC* mutations. Nuclear β‐catenin expression is also less frequent in G‐type than in intestinal‐type lesions [3]. Herein, we report a duodenal gastric foveolar‐type hyperplastic polyp with *GNAS* and *KRAS* mutations, highlighting the importance of histopathological and molecular evaluation even in lesions without cytological dysplasia. Although previous studies documented *GNAS* and *KRAS* mutations in G‐type lesions, including case series [1, 3], detailed assessments of a single histologically benign lesion are rare. This rarity underscores the novelty and clinical significance of this case.

## 2 Case Report

**FIGURE 1:**
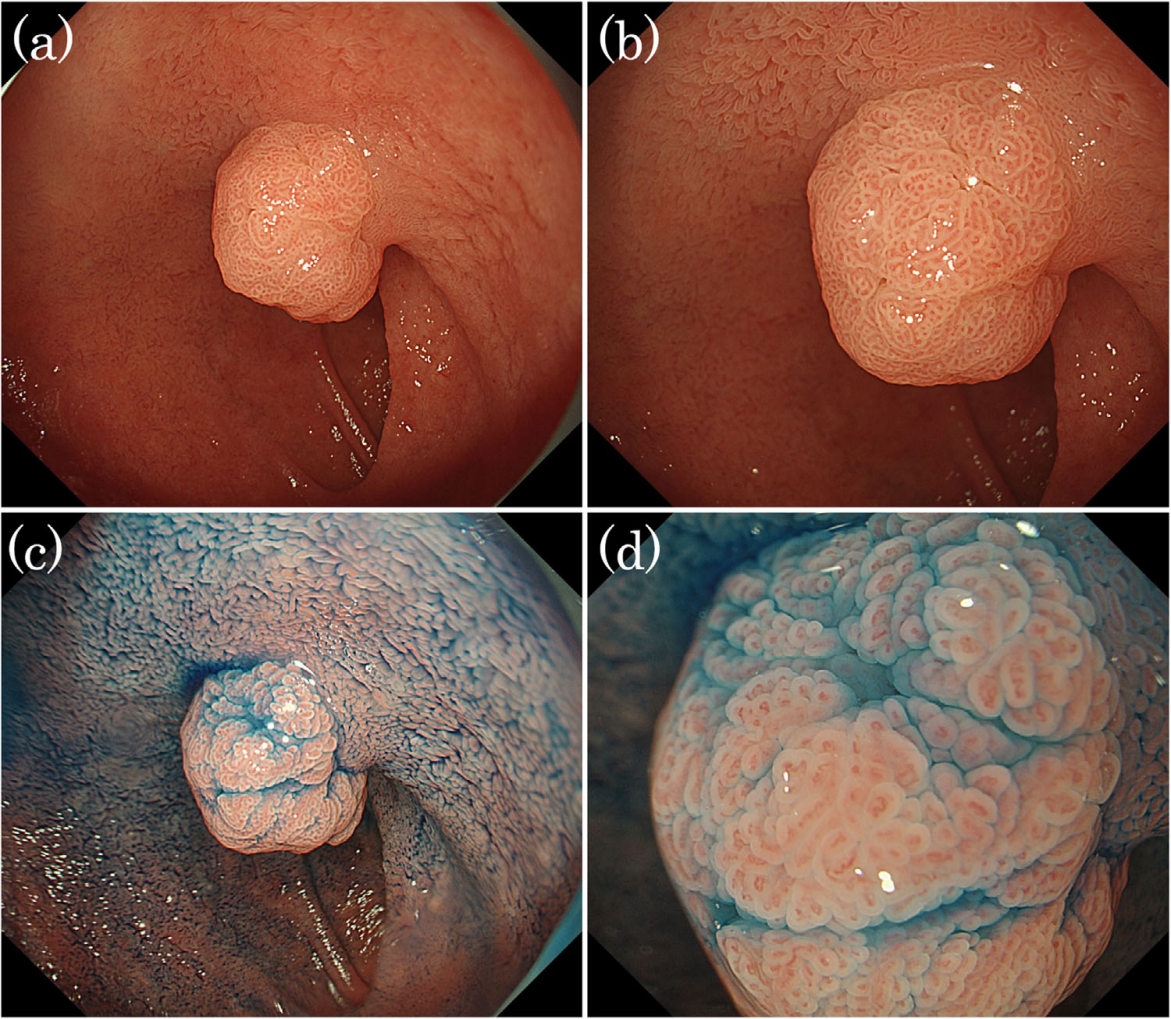
Endoscopic findings. (a) Distant view under conventional white‐light endoscopy showing a mildly erythematous, protruded 10‐mm lesion at the superior duodenal angle. No evidence of gastric foveolar metaplasia or heterotopic gastric mucosa was observed in the surrounding duodenal mucosa. (b) Close‐up view corresponding to Figure 1a, highlighting the lesion surface structure and showing a papillary and granular mucosal pattern. (c) Distant view using indigo carmine chromoendoscopy showing a papillary or granular mucosal pattern. No evidence of gastric foveolar metaplasia or heterotopic gastric mucosa was observed in the surrounding duodenal mucosa. (d) Close‐up view of Figure 1c, demonstrating papillary or granular mucosal pattern.

**FIGURE 2:**
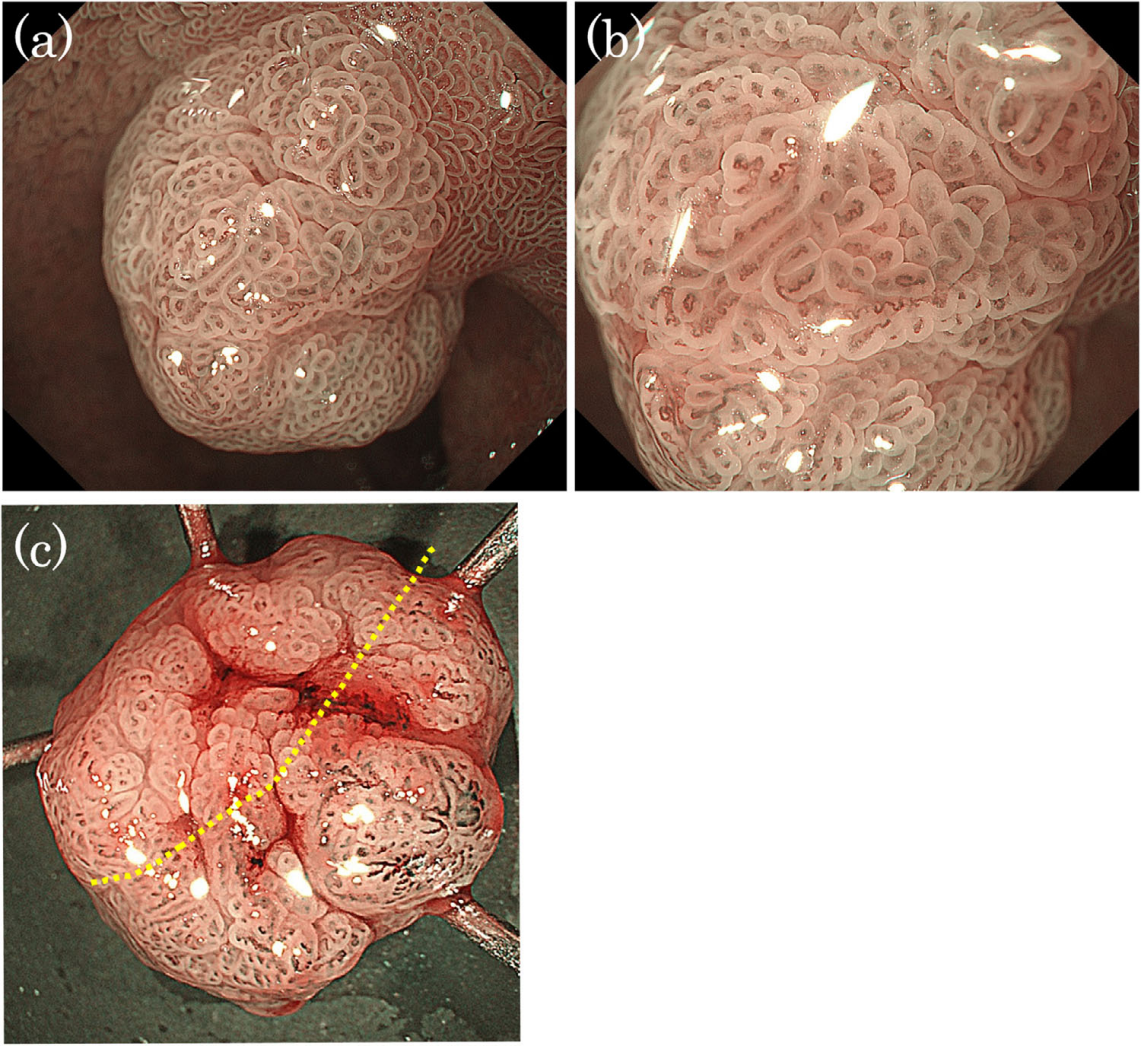
Endoscopic findings and resected specimen. (a) Distant view with narrow‐band imaging. No gastric foveolar metaplasia or heterotopic gastric mucosa was observed in the surrounding duodenal mucosa. (b) Magnified view of Figure 2a, revealing a slightly irregular papillary and granular mucosal pattern with a closed loop structure. The marginal epithelium was uniformly widened to approximately twice the width of the adjacent mucosa. No white opaque substance was observed. (c) Freshly resected specimen after endoscopic mucosal resection.

**FIGURE 3:**
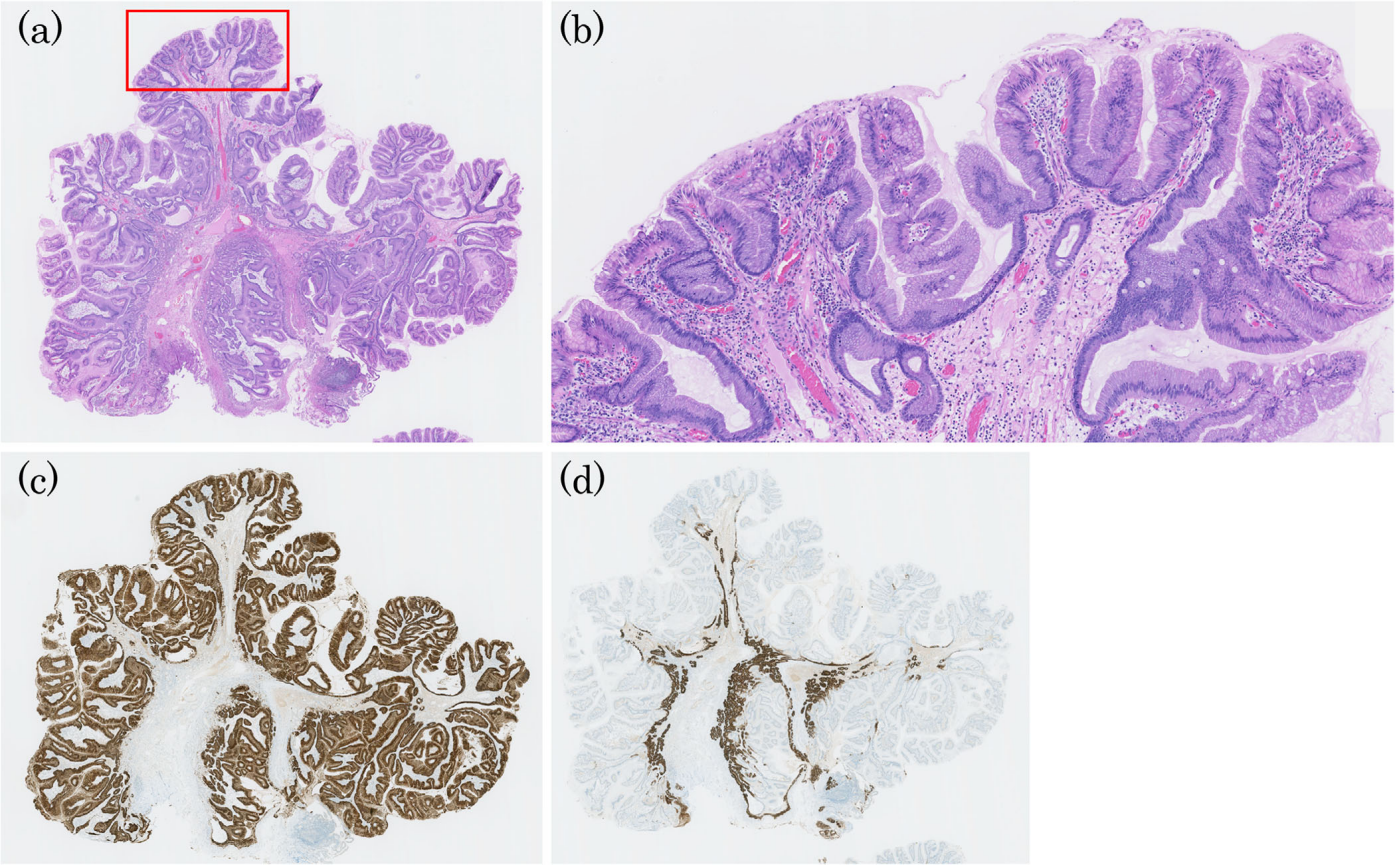
Histopathological and immunohistochemical findings of the resected specimen shown in Figure 2c. (a) Hematoxylin and Eosin staining of the cut surface (yellow dotted line in Figure 2c) showing monotonous papillary and villous structures of gastric‐type epithelium. There was no evidence of Brunner's gland hyperplasia or active inflammation. (Hematoxylin and Eosin staining, 40× magnification). (b) Close‐up view of the red‐outlined area in Figure 3a (Hematoxylin and Eosin staining, 200× magnification) showing gastric foveolar‐type epithelial cells with abundant cytoplasmic mucin. No cytological or architectural dysplasia was observed. (c) MUC5AC: Diffuse and strong positivity was observed on the surface and papillary epithelium. (d) MUC6: Expression was limited to a small number of deep glands.

**FIGURE 4:**
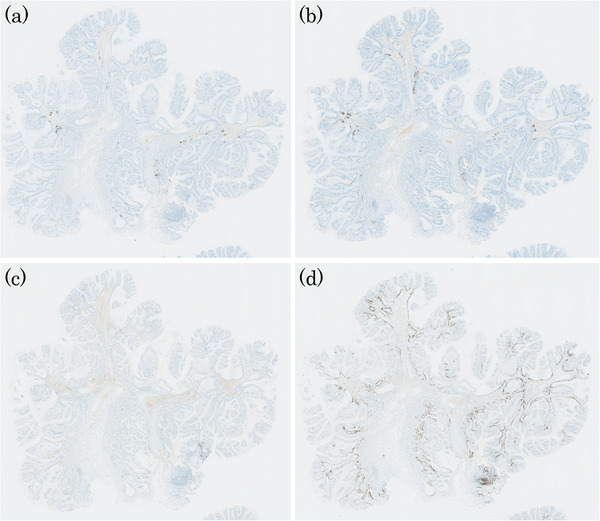
Additional immunohistochemical features. (a) Proton pump α: Few positive cells. (b) Proton pump β: Few positive cells. (c) Pepsinogen‐I: Rare positive cells. (d) Ki‐67: Positive cells confined to the basal portions of the pits.

A man in his 50s was referred to our hospital after a protruded lesion was detected in the duodenum during routine esophagogastroduodenoscopic screening. He had a history of successful *Helicobacter pylori* eradication and was not taking any prescribed medication at the time. The patient's family history was unremarkable. Endoscopy revealed a mildly erythematous, protruded lesion measuring 10 mm at the superior duodenal angle (Figure 1a,b). Indigo carmine chromoendoscopy clearly visualized the mucosal surface of the lesion, revealing a papillary or granular mucosal pattern (Figure 1c,d). No GFM or HGM was observed in the background duodenal mucosa on conventional and magnifying endoscopic observation with narrow‐band imaging (ME‐NBI). ME‐NBI revealed papillary and granular mucosal patterns with mild irregularities. However, the marginal epithelium appeared uniformly widened to approximately twice the width of the adjacent mucosa, consistent with an enlarged marginal epithelium (EME; Figure 2a,b). It was diagnosed as a non‐neoplastic lesion (NNL) by Nakayama et al.’s criteria [4], but due to its 10‐mm diameter and a slightly irregular mucosal pattern, endoscopic mucosal resection was performed (Figure 2c). The resected lesion measured 10 mm × 8 mm. Histopathologically, it was diffusely composed of gastric foveolar‐type epithelial cells with abundant cytoplasmic mucin arranged in papillary and villous architecture. The overall appearance was rather monotonous, without cytological dysplasia or architectural complexity, and thus not suggestive of neoplasia. There was no evidence of Brunner's gland hyperplasia or active inflammation (Figure 3a,b). Immunohistochemically, the surface and papillary epithelial cells were diffusely positive for MUC5AC (Figure 3c) and negative for MUC2 and CD10 (figures not shown), whereas MUC6 expression (Figure 3d) was limited to a small number of deep glands, indicating no aberrant differentiation. Occasional proton pump‐positive cells were observed, whereas pepsinogen I‐positive cells were extremely rare, indicating HGM was unlikely (Figure 4a–c). Ki‐67 positive cells were restricted to the bottom of the pits (Figure 4d). Genomic DNA from the duodenal lesion was subjected to whole‐exome sequencing using a SureSelectXT Human All Exon V6 kit (Agilent Technologies, Inc., Santa Clara, CA, USA) and an Illumina NovaSeq 6000 platform (Illumina, Inc., San Diego, CA, USA). Sequencing data were processed, and variants were called using the DRAGEN Bio‐IT platform v4.2.7 (Illumina, Inc.), with annotation performed using standard public databases. Genetic analysis identified activating mutations in both *GNAS* (c.602G>A, p.R201H) and *KRAS* (c.35G>A, p.G12D). No pathogenic alterations were identified in other cancer‐related genes (*BRAF*, *APC*, and TP53) besides *GNAS* and *KRAS*. Collectively, these findings support the diagnosis of a gastric foveolar‐type hyperplastic polyp of the duodenum harboring *GNAS* and *KRAS* mutations.

## 3 Discussion

*GNAS* and *KRAS* mutations are frequently observed in pyloric gland adenomas and gastric‐type adenocarcinomas and are considered to represent characteristic molecular alterations in G‐type NADETs [1]. GFM and HGM were traditionally considered reactive or congenital; mucosal injury near Brunner's glands may induce regeneration with MUC5AC‐positive epithelium, resulting in GFM [5]. NADETs can be classified into two main categories according to the phenotype: intestinal or gastric [6]. While the main stepwise progression of duodenal carcinogenesis involves an adenoma–carcinoma sequence, similar to colorectal tumors, particularly in tumors arising distally, NADETs arising proximal to the ampulla of Vater are frequently associated with G‐type histology and reportedly display higher potential for malignancy than those that arise distally [3, 6]. G‐type NADETs often present as elevated lesions, as was the case in our patient [2, 3]. Regarding mucin phenotype continuity, GFM and HGM are considered plausible precursor lesions for G‐type NADETs that arise in the proximal duodenum. A recent study reported *GNAS* and *KRAS* mutations in some GFM and HGM lesions, suggesting that they may represent early proliferative precursors with neoplastic potential rather than merely reactive or congenital changes [1, 2].

*GNAS* encodes the stimulatory G‐protein alpha subunit, which mediates intracellular signaling via G‐protein‐coupled receptors [3]. Activating *GNAS* mutations have been identified in various gastrointestinal neoplasms, including pyloric gland adenomas, duodenal gastric‐type adenocarcinomas, pancreatic intraductal papillary mucinous neoplasms, and colorectal villous adenomas [3]. *KRAS* mutations, which often co‐occur with *GNAS* mutations, have also been implicated in neoplastic progression [1].

Histopathologically, the lesion in the present case did not exhibit cytological dysplasia or complex architecture. However, closer examination revealed a monotonous proliferation of MUC5AC‐positive gastric foveolar‐type epithelial cells, with minimal inflammatory infiltration. GFM was not observed in continuity with the Brunner's glands or as separate mucosal components, and no HGM was identified. Therefore, the lesion was diagnosed as a gastric foveolar‐type hyperplastic polyp, distinct from GFM, because it lacked continuity with Brunner's gland hyperplasia and showed no significant inflammation. Matsueda et al. reported that G‐type NADETs were accompanied by GFM in 77% and HGM in 45% of cases [7]. In the present case, neither GFM nor HGM was identified by ME‐NBI or histopathological examination. Nevertheless, the detection of *GNAS* and *KRAS* mutations, previously reported in cases of both GFM and HGM, raises the possibility that this lesion originated from undetected or regressed GFM or HGM, or developed through an unidentified pathway independent of these precursor conditions. Matsubara et al. demonstrated that G‐type lesions, including GFM and HGM, frequently harbor the same mutations found in G‐type NADETs.^1^ Reported frequencies of *GNAS* mutations in GFM and HGM are 41% and 28%, respectively, while *KRAS* mutations occur in 26% and 2% [1]. *GNAS* and *KRAS* mutations have also been reported in 32% and 47%, respectively, of duodenal gastric‐type adenocarcinomas [1]. Our findings align with recent data indicating that *GNAS* mutations are characteristic of G‐type lesions and may coexist with *KRAS* mutations, whereas *APC* alterations characterize intestinal‐type lesions [3]. Although GFM and HGM are relatively common (19% [8] and 7.9% [9], respectively), G‐type NADETs remain rare [3, 6, 7]. This discrepancy suggests that while *GNAS* and *KRAS* mutations may represent early molecular alterations in precursor lesions, they are insufficient on their own to drive neoplastic transformation. Their frequent detection in NNL, such as GFM and HGM, compared with the low incidence of G‐type NADETs, suggests the requirement of additional molecular or environmental factors for malignant progression. Further investigation is warranted to clarify their role in tumorigenesis and identify true precursors to neoplastic lesions.

Endoscopically, the lesion showed clear demarcation, a closed‐loop structure, and EME, with only slight mucosal irregularity. According to Nakayama et al.’s classification algorithm for superficial NADETs, it was diagnosed as NNL [4]. The EME likely reflects the abundant mucinous cytoplasm characteristic of gastric foveolar‐type epithelium. This feature may represent a morphological correlate of enhanced secretory activity via constitutive cAMP–PKA pathway activation by mutant GNAS, as previously reported [10]. A biopsy alone would likely have appeared benign. However, no consensus exists on which G‐type lesions warrant resection, and overtreatment must be avoided. Further evidence is required to establish clear management criteria. This case highlights the potential value of incorporating molecular profiling with endoscopic and histological findings when determining management strategies.

## Ethics Statement

Institutional Review Board approval was not required for this study.

## Consent

Informed consent was obtained from the patient for publication of this case report.

## Conflicts of Interest

The authors declare no conflicts of interest.
